# Sequencing of complete mitochondrial genomes confirms synonymization of *Hyalomma asiaticum asiaticum* and *kozlovi*, and advances phylogenetic hypotheses for the Ixodidae

**DOI:** 10.1371/journal.pone.0197524

**Published:** 2018-05-16

**Authors:** Zhi-Qiang Liu, Yan-Feng Liu, Nuer Kuermanali, Deng-Feng Wang, Shi-Jun Chen, Hui-Ling Guo, Li Zhao, Jun-Wei Wang, Tao Han, Yuan-Zhi Wang, Jie Wang, Chen-Feng Shen, Zhuang-Zhi Zhang, Chuang-Fu Chen

**Affiliations:** 1 College of Animal Science and Technology, Shihezi University, Shihezi, Xinjiang Uygur Autonomous Region, China; 2 Institute of Veterinary Medicine, Xinjiang Academy of Animal Science, Urumqi, Xinjiang Uygur Autonomous Region, China; 3 School of Medicine, Shihezi University, Shihezi, Xinjiang Uygur Autonomous Region, China; University of Minnesota, UNITED STATES

## Abstract

Phylogeny of hard ticks (Ixodidae) remains unresolved. Mitochondrial genomes (mitogenomes) are increasingly used to resolve phylogenetic controversies, but remain unavailable for the entire large *Hyalomma* genus. *Hyalomma asiaticum* is a parasitic tick distributed throughout the Asia. As a result of great morphological variability, two subspecies have been recognised historically; until a morphological data-based synonymization was proposed. However, this hypothesis was never tested using molecular data. Therefore, objectives of this study were to: 1. sequence the first *Hyalomma* mitogenome; 2. scrutinise the proposed synonymization using molecular data, i.e. complete mitogenomes of both subspecies: *H*. *a*. *asiaticum and kozlovi*; 3. conduct phylogenomic and comparative analyses of all available Ixodidae mitogenomes. Results corroborate the proposed synonymization: the two mitogenomes are almost identical (99.6%). Genomic features of both mitogenomes are standard for Metastriata; which includes the presence of two control regions and all three "Tick-Box" motifs. Gene order and strand distribution are perfectly conserved for the entire Metastriata group. Suspecting compositional biases, we conducted phylogenetic analyses (29 almost complete mitogenomes) using homogeneous and heterogeneous (CAT) models of substitution. The results were congruent, apart from the deep-level topology of prostriate ticks (*Ixodes*): the homogeneous model produced a monophyletic *Ixodes*, but the CAT model produced a paraphyletic *Ixodes* (and thereby Prostriata), divided into Australasian and non-Australasian clades. This topology implies that all metastriate ticks have evolved from the ancestor of the non-Australian branch of prostriate ticks. Metastriata was divided into three clades: 1. Amblyomminae and Rhipicephalinae (*Rhipicephalus*, *Hyalomma*, *Dermacentor*); 2. Haemaphysalinae and Bothriocrotoninae, plus *Amblyomma sphenodonti*; 3. *Amblyomma elaphense*, basal to all Metastriata. We conclude that mitogenomes have the potential to resolve the long-standing debate about the evolutionary history of ticks, but heterogeneous evolutionary models should be used to alleviate the effects of compositional heterogeneity on deep-level relationships.

## Introduction

Ticks (Chelicerata: Ixodida) are one of the medically most important groups of arthropods; as obligate hematophagous parasites of terrestrial vertebrates, they are vectors of several very important diseases. Despite their medical and veterinary importance, genetic resources for ticks remain relatively limited and our understanding of tick evolution merely fragmental [1–5]. Among the three extant families of ticks, only two are of importance for public health: Ixodidae (the hard ticks) and Argasidae (the soft ticks) [6]. Due to unsuitability of morphological traits for the task and limited genetic resources currently available, phylogeny and taxonomy of Ixodidae remain unresolved. On the basis of morphological differences, the hard ticks are divided into two groups: Prostriata—containing only the Ixodinae subfamily, and Metastriata—containing a debated number of genera (around 13) classified into a debated number of subfamilies (4 or 5) [5,7–9]. Among the metastriate ticks, species belonging to the large (27 currently recognized species) *Hyalomma* Koch 1844 genus are distributed from tropical Africa to Siberia [10,11]. Species of this genus infest mammals, birds and reptiles; and they are vectors of several important viruses and rickettsial organisms [12]. *Hyalomma asiaticum* Schulze and Schlottke 1930 is a tick species common almost in the entire Asia, which transmits a number of different diseases and exhibits great morphological variability [10,13]. This has historically caused a number of taxonomic controversies, including proposals of a varying number of subspecies [10]. Two of these subspecies, *H*. *a*. *asiaticum* and *kozlovi*, were considered valid until a relatively recent study proposed their synonymization on the basis of morphological characters [10]. However, this hypothesis was never tested using molecular data.

Although the phylogeny of Ixodida has been studied using a number of molecular markers, both mitochondrial and nuclear [9,14,15], the resolution that these single molecular markers provide appears to be too low to unequivocally resolve the evolutionary history of this group of animals. Mitochondrial genomes (mitogenomes) carry a large amount of data, which makes them capable of providing much higher resolution than traditionally used morphological and single-gene molecular markers, and therefore mitochondrial phylogenomics is increasingly used to address controversial phylogenetic relationships [16–18]. Mitochondrial phylogenomics has also been used to study the phylogeny of ticks [3,19–21], but its applicability remains hampered by the unavailability of mitogenomes for many large taxonomic categories, including the entire *Hyalomma* genus.

As it appears that a much larger amount of molecular data will have to be available in order fully resolve the evolutionary history of hard ticks, the objective of this study was to sequence the first *Hyalomma* mitogenome. Additionally, as Apanaskevich and Horak [10] did not use any molecular data to support their synonymization of two *H*. *asiaticum* subspecies (*asiaticum* and *kozlovi*), we have set out to corroborate their proposition using mitogenomic data. To achieve this, we sampled specimens morphologically corresponding to the two subspecies [6,10,22] and sequenced their mitogenomes. Following this, we conducted comparative and phylogenomic analyses using all available hard tick mitogenomes.

## Results and discussion

The two mitogenomes are almost identical, with identity of 99.6% (merely 48 variable sites were found), and only four gaps in the alignment. Previously, sequence difference between two complete mitogenomes of a closely related species, *Rhipicephalus sanguineus* (one specimen from China and one from the USA), was found to be 11.23%, thereby leading the authors to propose that these are two different species [20]. We can therefore make exactly the opposite conclusion, that the sequencing of these two mitogenomes has corroborated the morphology-based hypothesis [10] that *Hyalomma asiaticum asiaticum* (S1 Fig) and *H*. *a*. *kozlovi* (S2 Fig) should be synonymized to *Hyalomma asiaticum*.

### Genome architecture

The complete mitochondrial genome of *H*. *a*. *asiaticum* (Haa; GenBank accession number: MF101817) is 14 720 bp-long, whereas that of *H*. *a*. *kozlovi* (Hak; MF101818) is four bp longer—14 724. Both mitogenomes possess the standard 13 protein-coding genes, two rRNA genes (16S and 12S), 22 tRNA genes and two control regions. The mean AT and GC skews are -0.027 and -0.144 respectively. All these characteristics are standard for ticks (S1 File) [3,5,9,20]. The A+T content (78.1% Haa, 78.2% Hak) is also average for this group of animals: metastriate ticks have the A+T content between 75 and 80%, whereas soft ticks have a somewhat lower A+T content of 70–75% [3,5,9,20].

**Fig 1:**
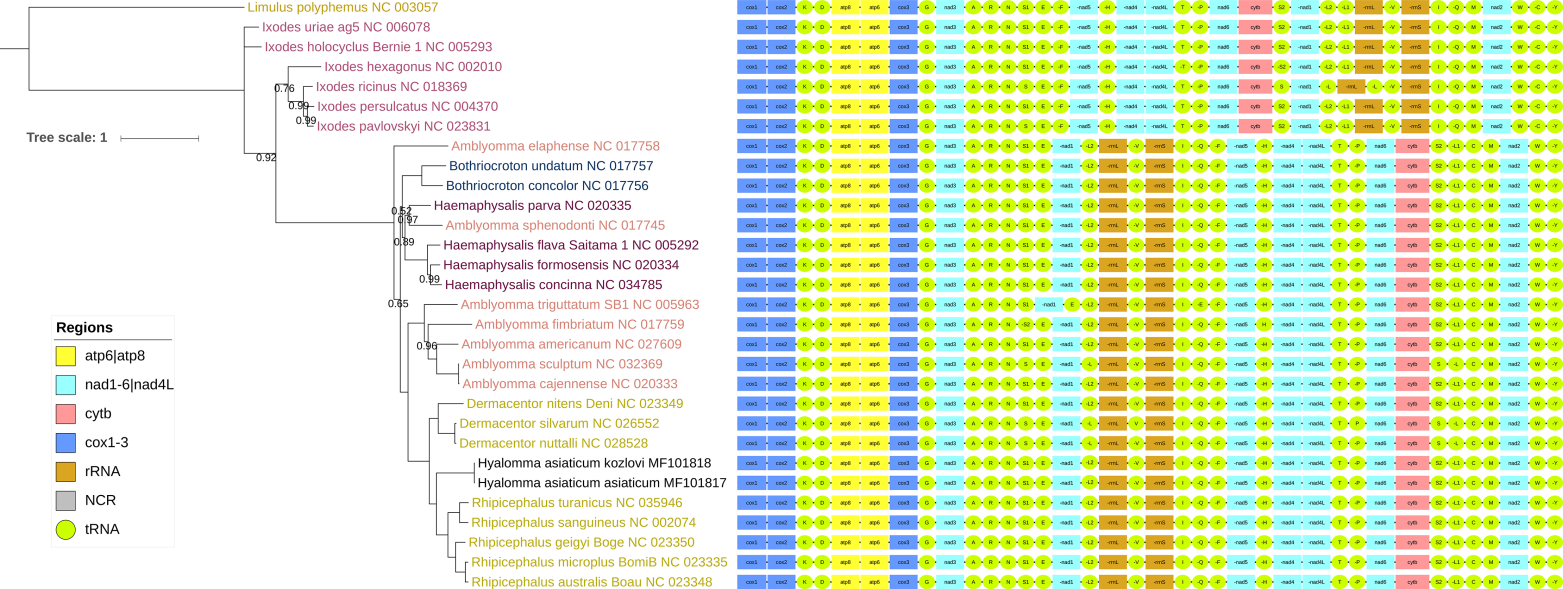
Mitochondrial phylogenomics and mitogenomic architecture of the Ixodidae family. Phylogenetic dendrogram was constructed using nucleotide sequences of almost complete 29 available Ixodidae mitogenomes. Heterogeneous CAT model implemented in PhyloBayes was used to conduct the Bayesian inference analysis. *Limulus polyphemus* is the outgroup. Scale bar corresponds to the estimated number of substitutions per site. Only the Bayesian probability values lower than 1.0 are shown next to corresponding nodes. Mitogenomic architecture is shown to the right of the corresponding sequences (the legend is incorporated in the figure). GenBank accession numbers are shown next to species names. Font colours correspond to subfamilies (according to the GenBank taxonomy), with full details available in the S1 File (supplementary data).

In terms of gene order and strand distribution, the ancestral arthropod architecture (*Limulus polyphemus* in Fig 1) has remained unchanged for over 400 million years in the lineages leading to the prostriate ticks (two *Ixodes* clades in Fig 1) [8,23]. In metastriate ticks, a block of seven genes (*nad1*-trnL2-rrnL-trnV-rrnS-trnI-trnQ) was translocated, some tRNAs were rearranged (Fig 1), and two large non-coding (control) regions are usually present [7–9,23]. However, gene order and strand distribution within this group (metastriate ticks), which includes the two new mitogenomes as well, are perfectly conserved (Fig 1). The only exception is *Amblyomma trigutatum*, where *nad1* and tRNA-Glu have switched places; but as this mitogenome remains unpublished, we don’t exclude a possibility of an annotation mistake (we are quite confident that tRNA-Ser2 on the minority strand in *A*. *fimbriatum*, where tRNA-Ser1 should be on the majority strand, is an annotation artefact).

We found 13 intergenic regions (IGRs) and nine gene overlaps. IGRs ranged from 1 to 21 bp in size, with the longest located between the tRNA-Gln and tRNA-Phe genes (Table 1). The overlaps ranged from 1 to 8 bp, with the largest found between tRNA-Tyr/*cox1* (8 bp), *atp6*/*atp8* (7 bp), and *nad4*/*nad4L* (7 bp). Although the possibility of annotation artefacts should not be excluded, the comparison of homologs in related species (S1 File) and almost identical numbers and sizes of overlaps and IGRs in some related species [20], do not provide any indication of this. Apart from two large overlaps, *atp6*/*atp8* and *nad4*/*nad4L*, all other overlaps involved tRNA genes, which is common, and believed to be a consequence of lesser evolutionary constraints on tRNA sequences [24]. Identical overlaps of two PCGs (*atp6*/*atp8* and *nad4*/*nad4L*, both 7 bp) have been described in another metastriate tick, *A*. *sculptum* [21], but metastriate ticks belonging to the *Rhipicephalus* genus appear to exhibit only an identical (7 bp) *atp6*/*atp8* overlap [20]. Both overlaps include very small genes (*atp8* = 162 bp, *nad4L* = 276 bp), which appear to be under lesser evolutionary constraints, as *atp8* is often absent from mitogenomes [25], whereas the *nad4*/*nad4L* overlap is common in mitogenomes of many different groups of animals [17,18,26–28]. Although annotation of the *nad1* gene is usually very difficult in hard ticks, often producing unusually large gene overlaps [21,29], *nad1* genes in the studied two mitogenomes are very similar to closely related *Rhipicephalus* orthologs (S1 File), with an overlap of only 4 bp with the neighbouring tRNA-Glu (Table 1).

**Table 1:** The organization of *Hyalomma asiaticum asiaticum* (Haa) and *kozlovi* (Hak) mitochondrial genomes.

Gene	Haa			Hak			IGR	Codon		Strand
	From	To	Size	From	To	Size		Start	Stop	
tRNA-Met	1	65	65	1	65	65				+
*nad2*	66	1028	963	66	1028	963		ATT	TAA	+
tRNA-Trp	1029	1089	61	1029	1089	61				+
tRNA-Tyr	1088	1148	61	1088	1148	61	-2			-
*cox1*	1141	2679	1539	1141	2679	1539	-8	ATT	TAA	+
*cox2*	2683	3355	673	2683	3355	673	3	ATG	T--	+
tRNA-Lys	3356	3421	66	3356	3421	66				+
tRNA-Asp	3422	3482	61	3422	3482	61				+
*atp8*	3484	3645	162	3484	3645	162	1	ATG	TAA	+
*atp6*	3639	4304	666	3639	4304	666	-7	ATG	TAA	+
*cox3*	4309	5086	778	4309	5086	778	4	ATG	T--	+
tRNA-Gly	5087	5147	61	5087	5147	61				+
*nad3*	5148	5489	342	5148	5489	342		ATT	TAA	+
tRNA-Ala	5489	5549	61	5489	5549	61	-1			+
tRNA-Arg	5556	5619	64	5556	5619	64	6			+
tRNA-Asn	5618	5679	62	5618	5680	63	-2			+
tRNA-Ser-1	5677	5732	56	5678	5734	57	-3			+
tRNA-Glu	5738	5800	63	5740	5802	63	5			+
tick-box-1	5803	5819	17	5805	5821	17				-
*nad1*	5797	6736	940	5799	6738	940	-4	ATT	T--	-
tRNA-Leu-2	6737	6799	63	6739	6801	63				-
rrnL	6800	7989	1190	6802	7992	1191				-
tick-box-2	6805	6823	17	6807	6823	17				-
tRNA-Val	7990	8048	59	7993	8051	59				-
rrnS	8049	8747	699	8052	8750	699				-
CR1	8748	9050	303	8751	9053	303				
tRNA-Ile	9051	9113	63	9054	9116	63				+
tRNA-Gln	9117	9181	65	9120	9184	65	3			-
tick-box-3	9184	9200	17	9187	9203	17				+
tRNA-Phe	9203	9262	60	9206	9265	60	21			-
*nad5*	9263	10903	1641	9266	10906	1641	0	ATA	TAA	-
tRNA-His	10922	10982	61	10925	10985	61	18			-
*nad4*	10988	12304	1317	10991	12307	1317	5	ATG	TAG	-
*nad4L*	12298	12573	276	12301	12576	276	-7	ATG	TAA	-
tRNA-Thr	12576	12636	61	12579	12640	62	2			+
tRNA-Pro	12637	12699	63	12641	12703	63				-
*nad6*	12702	13136	435	12706	13140	435	2	ATA	TAA	+
*cytb*	13140	14219	1080	13144	14223	1080	3	ATG	TAA	+
tRNA-Ser-2	14220	14287	68	14224	14291	68				+
tRNA-Leu-1	14287	14355	69	14291	14359	69	-1			-
CR2	14356	14661	306	14360	14665	306				+
tRNA-Cys	14662	14718	57	14666	14722	57				+

IGRs, codons and strand distribution are identical between the two mitogenomes. IGR is intergenic region, where a negative value indicates an overlap.

Gene sizes were identical between the two mitogenomes, and relatively standard for the entire Ixodidae group. Generally, gene sizes are highly conserved among the available Ixodidae mitogenomes, with most genes exhibiting a size-window smaller than 10 bp; exceptions are only *nad2* and *nad5* with approximately 30 bp size-windows (S1 File B worksheet). Regarding the two studied mitogenomes, the only outlier is *nad5* gene (1641 bp), which is smaller than in other available orthologs, with only the *Amblyomma americanum* [3] ortholog exhibiting a similar size (1642 bp). Both studied mitogenomes used identical start and termination codons. The start codons used were ATG (6), ATA (4), and ATT (3), whereas the termination codons were TAA (9), T--(3) and TAG (1), all of which are standard for ticks [9,20]. Intriguingly, *atp8* used ATG, which is non-standard for this group of animals; it was reported only in *Amblyomma americanum* [3] and *Ixodes hexagonus* [7] among the available Ixodidae mitogenomes, whereas the rest of the species use ATT, ATC or ATA (S1 File, worksheet B). As observed in other ticks, two AT-rich or non-coding regions were found (CR1 and CR2, Table 1). Both are typical in terms of size (303 and 306 bp, respectively) and location (CR1—between the rrnS and tRNA-Ile, CR2 between tRNA-Leu-1 and tRNA-Cys) [7,9,20].

Tick-Boxes are two to three degenerate 17 bp-long motifs (ttgyrtchwwwtwwgda) discovered in tick mitogenomes, which are believed to be post-transcriptional regulatory elements, and might also have been involved in genome rearrangements [29]. Although the third box is not present in all metastriate ticks [3,29], all three boxes were found in the two studied mitogenomes (Table 1) exactly in the positions described before [29]. The six motifs were highly conserved; only two different sequences were found, differing by only a T↔A mutation: TTGCATCATTTTTTGGA (both Tick-Boxes-1 and Haa Tick-Box-2) and TTGCATCAATTTTTGGA (both Tick-Boxes-3 and Hak Tick-Box-2).

### Phylogeny

Overview of published phylogenetic studies reveals a notable variability in the topology produced (both in Prostriata and Metastriata clades) depending on the dataset and methodology used [3,5,8,9,19,20]. As differences are particularly pronounced between mitochondrial and nuclear datasets, we suspected that this might be an indication of the existence of either compositional heterogeneity in the mitogenomes of ticks, or possibly even mitochondrial introgression in the evolutionary history of ticks [30]. Although recent evidence shows that the latter is more widespread than previously thought [31–33], previously observed effects of the third codon position exclusion from mitochondrial PCGs [5,9] suggest that compositional heterogeneity is the more likely cause for this inconsistency. Mitochondrial genomes of some groups of animals, which also includes some Arthropoda, often exhibit compositional heterogeneity, or non-constant equilibrium nucleotide frequencies across different lineages, which is a major driver of artificial clustering (long-branch attraction) in phylogenetic analysis [34–36]. Therefore, homogeneous models of substitution, where all sites evolve under the same substitution process and constantly through time, may not be suitable for mitochondrial phylogenomics studies in ticks. Among the strategies designed to minimise these biases, site-heterogeneous mixture model (CAT), which allows flexible probabilities of the nucleotide replacement equilibrium frequencies, is considered to be the most effective [36]. Therefore, we have decided to test both the standard homogeneous model and a non-standard heterogeneous model on our dataset. The results they produced were mostly congruent, apart from the deep-level topology of prostriate ticks (*Ixodes*): the homogeneous model (S3 Fig) produced a monophyletic *Ixodes* subdivided into two sister-clades (Australasian and non-Australasian), whereas the heterogeneous CAT model produced a paraphyletic *Ixodes* (and thereby Prostriata) with very high nodal support (Fig 1). Although the monophyly of *Ixodes* has been questioned before [5,8,19,37–40], a majority of morphological and nuclear, and all mitochondrial (including the amino acid sequences) datasets, produced monophyletic *Ixodes* [3,5,8,9,19,20,39,41]. The fact that ours is the first study relying on mitochondrial data to produce paraphyletic *Ixodes* is in perfect agreement with the observation that impact of compositional heterogeneity is much more pronounced in deep-level phylogenies [36]. Paraphyletic *Ixodes* genus was produced in several studies using concatenated nuclear 18 and 28S gene datasets [5,8,19,37], but in these analyses non-Australian *Ixodes* group was basal to Australian *Ixodes*. The topology retrieved in our study, where Australian *Ixodes* clade was basal to the non-Australian *Ixodes* clade. Therefore, our results produced using the CAT heterogeneous model imply that all metastriate ticks have evolved from the ancestor of the non-Australian branch of prostriate ticks after it became separate from the Australian branch of Prostriata. These findings are very interesting from the aspect of a previously proposed hypothesis that ticks have originated in the Australasian region of Gondwanaland [38,40,42] (see [2] for detailed discussion). However, this intriguing hypothesis would have to be tested very carefully (regarding the methodology) using a much larger amount of both nuclear and mitochondrial molecular data.

The rest of the topology did not produce novel findings, but it might help resolve a number of phylogenetic controversies. Metastriata was divided into two major clades: 1. Amblyomminae, Rhipicephalinae (*Rhipicephalus* and *Dermacentor* genera), and Hyalomminae; and 2. Haemaphysalinae and Bothriocrotoninae, plus *Amblyomma sphenodonti*. It should be noted that the taxonomy we used here is the one currently used for the GenBank entries. Our topology indicates that clade 1 should be subdivided into Amblyomminae (*Amblyomma*) and Rhipicephalinae (*Rhipicephalus*, *Hyalomma*, and *Dermacentor* genera). This is in agreement with some [3,5,9,19,41], and disagreement with other [8,20], previous studies, based on both nuclear and mitochondrial data. Unlike most previous studies [3,5,8,9,19], our topology does not support the existence of a particularly deep split between Haemaphysalinae and Bothriocrotoninae. However, our results do support the previously noted [5,19] deep division of the *Haemaphysalis* genus, and provides further support [5,19,20] for *A*. *elaphense* being basal to the rest of the Metastriata (albeit with a relatively low nodal support). However, the positions of these two species are extremely variable [5,9], their morphology still places them within their respective genera, mitochondrial introgression may have occurred in their evolutionary history, and we show here that analyses based on mitochondrial data may be affected by compositional heterogeneity; so these findings would have to be thoroughly supported by nuclear data before taxonomic changes can be proposed with confidence.

Intriguingly, most of the studies using 18S or concatenated nuclear 18S/28S datasets produced a paraphyletic *Rhipicephalus* (with *Hyalomma* at the bottom of the clade) [5,8,9,19]; and 18S dataset produced a sister clade relationship between Haemaphysalinae and Rhipicephalinae [8]. As our analyses did not produce any of these artefacts, this might be an evidence of a higher phylogenetic resolution conferred by the large mitogenomic dataset. However, a larger number of *Rhipicephalus* and *Hyalomma* mitogenomes would have to be available to make that conclusion with certainty.

## Conclusions

As the absence of a sufficient number of sequenced mitogenomes is currently the foremost limiting factor for their application to infer the evolutionary history of ticks, we have sequenced the first mitogenome of two subspecies belonging to a large *Hyalomma* genus: *H*. *asiaticum asiaticum* and *kozlovi*. As the two sequenced mitogenomes are almost identical, our results corroborate the hypothesis of Apanaskevich and Horak [10] that the two subspecies should be synonymized. The results of our phylogenetic analysis imply that all metastriate ticks may have evolved from the ancestor of the non-Australian branch of prostriate ticks, but further analyses and more data shall be needed to corroborate this scenario. Although our results indicate that mitogenomes might have the potential to resolve the long-standing debate about the evolutionary history of ticks, the indication that compositional heterogeneity might affect the deep-level relationships strongly implies that future studies relying on the mitochondrial phylogenomics approach should combine homogeneous and heterogeneous evolutionary models in order to identify the long-branch attraction artefacts.

## Materials and methods

### Samples, identification, and DNA extraction

Adult *Hyalomma asiaticum asiaticum* (Schulze and Schlottke, 1929) ticks (S1 Fig) were collected from the skin of free-range grazing sheep in Qitai County, Xidi town, Xinjiang Uygur Autonomous Region, China. Qitai County is located in the north-eastern Xinjiang, north of the Tianshan Mountains, south-eastern margin of the Junggar basin (89°13’ - 91°22’ E, 42°25’ - 45°29’ N). Adult *Hyalomma asiaticum kozlovi* (Olenev, 1931) ticks (S2 Fig) were collected from the skin of free-range grazing sheep in Karamay city, Xiaoguai village, Xinjiang Uygur Autonomous Region. Karamay is located at the north-western margin of the Junggar basin (84°44’ - 86°01’ E, 44°07’ - 46°08’ N). Sampling was conducted from February to April 2015. Live ticks were collected into a sample box, kept alive in the lab for 3–5 days to ensure they were starved, and then stored in 75% ethanol at 4°C. Specimens were morphologically identified under a dissecting microscope as described before [6,10,22]. After washing in sterile water, DNA was isolated from one specimen of each putative subspecies using Aidlab DNA extraction kit (Aidlab Biotechnologies, Beijing, China). As the study involved unregulated parasitic invertebrates, no permits were required to retrieve and process the samples. We collected the ticks from privately owned sheep, free-range grazing on public land (arid grasslands, and semi-desert). For this, we collaborated with local veterinary departments, which took us to the farmers (sheep owners) to obtain the verbal agreement to collect the parasites. Therefore, no written permits were issued.

### Genome sequencing and assembly

Genomes were sequenced, assembled and annotated roughly following the procedure described before [18,28,43]. Ten primer pairs were designed (with approx. 100bp overlaps) to match the conserved regions of mitochondrial genes, and used to amplify and sequence the entire mitogenome (Table 2). Reaction volume of 50μL contained 5 U/μL of TaKaRa LA Taq polymerase (TaKaRa, Japan), 10×LATaq Buffer II, 2.5μM of dNTP mixture, 0.2–1.0μM of each primer, 60ng of DNA template, and PCR-grade H_2_O. Amplification was conducted under the following conditions: initial denaturation at 98°C for 2min, followed by 40 cycles at 98°C for 10s, 50°C for 15s, 68°C for 1 min/kb. If the product was not specific enough, PCR conditions were optimized: annealing temperature increased by two degrees and the number of cycles decreased to 35. PCR products were sequenced on an ABI 3730 automatic sequencer using Sanger method [44]. All obtained fragments were quality-proofed (electropherogram) and BLASTed [45] to confirm that the amplicon is the actual target sequence. Mitogenome was assembled stepwise manually with the help of DNAstar v7.1 [46] program, making sure that the overlaps were identical, and that no *numt*s [47] were incorporated into the sequence. DNAstar was also used to locate the putative ORFs for protein-coding genes (PCGs). Then we used BLAST and BLASTx to compare the putative ORFs with published nucleotide and amino acid sequences of related species, and manually determine the actual initiation and termination codon positions. Annotation of tRNAs was performed using tRNAscan [48] and ARWEN [49] tools, and the results checked manually. The annotation was recorded in Word documents, and parsed and extracted using MitoTool software [50]. MitoTool was also used to create GenBank submission files and tables with statistics for mitogenomes. Annotated mitogenomes are available from the GenBank via accession numbers MF101817 (Haa) and MF101818 (Hak).

**Table 2:** Primers used for amplification and sequencing of the mitochondrial genomes of *Hyalomma asiaticum asiaticum* and *kozlovi*.

Fragment No.	Gene or region	Primer name	Sequence (5’-3’)	Length (bp)
F1	*COX1*	LYF1	GCTGGGATATTAGGTCTTAG	1436
		LYR1	GAGTGTTCGGAGGGAGGGAA	
F2	*COX1-COX2*	LYF2	GACGTTATTCAGATTACCCTG	663
		LYR2	GGATAACAAGTTTGTTATCTG	
F3	*COX2*	LYF3	CTGATGAAACTTTTTCATCAC	356
		LYR3	GAAACTATGATTTGCACCAC	
F4	*COX2-COX3*	LYF4	GGACAATCCCATCACTTGGG	1462
		LYR4	TTAGGAGACAATCTTCTATG	
F5	*COX3*	LYF5	GATGTCTCACGAGAAGCAAG	442
		LYR5	GAAAGCCATGAAAACCAGTAG	
F6	*COX3-16S*	LYF6	CAATTATTCTTGGGATTAC	2402
		LYR6	GACCCTAAGAATTTGAAGATC	
F7	*16S*	LYF7	TACGCTGTTATCCCTAGAG	828
		LYR7	CGTACCTTTAGCATTAGGG	
F8	*16S-CYTB*	LYF8	GAAAAGAATTTCACATCTAAAG	5995
		LYR8	CCAAAAGGGTTTCTTGATCC	
F9	*CYTB*	LYF9	GCAATCCCATATATCGGTTC	370
		LYR9	GAAAGTACCATTCAGGTTG	
F10	*CYTB- COX1*	LYF10	CGATCATTTACCCTTATAGAC	2247
		LYR10	CGCCAATTATGATAGGTATAAC	

### Phylogenetic and comparative analyses

For the comparative and phylogenetic analyses we have retrieved all 27 currently (Nov. 2017) available Ixodidae mitogenomes (S1 File) from the NCBI’s non-redundant RefSeq database [51]. Sequence comparison (identity) between the two newly-sequenced mitogenomes was calculated using EMBL-EBI tools framework (Needle) [52]. For the phylogenetic analyses, following previous studies [9,19], a basal arthropod, *Limulus polyphemus* (NC_003057) [53], was used as the outgroup. Fasta files with nucleotide sequences for all 37 genes (12 PCGs, 2 rRNAs and 22 tRNAs) were extracted from GenBank files using MitoTool. Nucleotide sequences of protein-coding genes (PCGs) were aligned in batches (using codon-alignment mode) with MAFFT [54] integrated into BioSuite [55]. As described before [17], RNAs were aligned by Q-INS-i algorithm, which takes secondary structure information into account, incorporated into MAFFT-with-extensions software [56]. tRNAs Q, L1, L2, S1 and S2 were removed from the dataset for phylogenetic analysis because their annotation was not consistent in all mitogenomes. BioSuite was also used to concatenate these alignments and remove ambiguously aligned regions from the concatenated alignments by another plug-in program, GBlocks [57]. We used relaxed parameter settings [58] to maximize the amount of phylogenetic signal retained in the data: maximum/minimum number of sequences for a conserved/flank position = 16 (all), maximum number of contiguous non-conserved positions = 8, minimum length of a block = 10, allowed gap positions = “with half”. As a result, from the 15 408 positions in the original alignment, 12 690 (82%) remained. Selection of the most appropriate evolutionary model was computed using ModelFinder [59] and the IQ-TREE web interface [60]. All three algorithms (Akaike, Corrected Akaike, and Bayesian information criterion) produced identical best-fit model for a single partition: GY+F+I+G4 for nucleotide sequences of PCGs, TVM+G4 for rRNAs, and TIM2+I+G4 for tRNAs. This indicates that different parts of the mitogenome evolve under different rates. Furthermore, IQ-TREE performs a composition chi-square test for every sequence in the alignment, the purpose of which is to test for homogeneity of character composition: a sequence is denoted failed if its character composition significantly deviates from the average composition of the alignment [60]. Results of these analyses also indicated among-species compositional heterogeneity for concatenated PCGs (19 nucleotide sequences failed the composition chi^2^ test) and rRNAs (seven sequences failed the test). Regarding the concatenated tRNAs sequences, apart from *L*. *polyphemus*, all other sequences passed the chi^2^ test (S2 File). All these results indicate the existence of compositional heterogeneity, particularly among the PCG sequences, which can severely compromise the phylogenetic analysis [34,61]. Therefore, we have conducted the phylogenetic analysis using a program designed specifically to account for compositional heterogeneity, Phylobayes-MPI 1.7a [34,62], available from the beta version of the Cipres server (https://cushion3.sdsc.edu/portal2/tools.action) [63]. The CAT-GTR site mixture model implemented in PhyloBayes allows for site-specific rates of mutation, which is considered to be a more realistic model of amino acid evolution, especially for large multi-gene alignments [64]. Two MCMC chains were run after the removal of invariable sites from the alignment, and the analysis stopped when the conditions considered to indicate a good run (PhyloBayes manual) were reached: maxdiff < 0.1 and minimum effective size > 300. Other parameters were default (burnin = 500). Alignment file used for the analysis is supplemented (S3 File). We also conducted a phylogenetic analysis using a standard, homogeneous, evolutionary model: Maximum Likelihood algorithm (with 1000 bootstrap replicates) implemented in raxmlGUI [65,66], on Cipres webserver [63]. Phylograms and gene orders were visualized and annotated by iTOL [67] using several dataset files generated by MitoTool, as described previously [18].

## Supporting information

S1 Fig A typical *Hyalomma asiaticum asiaticum* specimen. (JPG)

S2 Fig A typical *Hyalomma asiaticum kozlovi* specimen. (JPG)

S3 Fig Mitochondrial phylogenomics of the Ixodidae family inferred using Maximum Likelihood analysis. Phylogenetic dendrogram was constructed using nucleotide sequences of almost complete 29 available Ixodidae mitogenomes. Maximum Likelihood analysis was conducted using a homogeneous evolutionary model implemented in RaxML. The branch of the outgroup, *Limulus polyphemus*, has been shortened. Scale bar corresponds to the estimated number of substitutions per site. Bootstrap values are shown next to the corresponding nodes. GenBank accession numbers are shown next to species names, with full details available in the S1 File (supplementary data).
(TIF)

S1 File Comparative analysis of Ixodidae mitogenomes. Worksheet A lists all species grouped by subfamilies (according to the GenBank taxonomy), with GenBank accession numbers, mitogenome sizes, base composition and skewness. Size, composition and skewness are also given for PCGs, rRNAs and tRNAs separately. Worksheet B lists gene sizes for all species, and their corresponding (putative) start and terminal codons. Species are represented by acronyms of their binomial scientific names. Worksheet C lists detailed base compositions of entire mitogenomes.
(XLSX)

S2 File Composition chi-square test. Composition chi-square test performed by IQ-TREE for concatenated protein-coding genes (PCGs), rRNAs and tRNAs. A sequence is denoted failed if its character composition significantly deviates from the average composition of the alignment.
(TXT)

S3 File Alignment used for the phylogenetic analyses. (TXT)
