# Supplementary figures and images for: Sequencing of complete mitochondrial genomes confirms synonymization of Hyalomma asiaticum asiaticum and kozlovi, and advances phylogenetic hypotheses for the Ixodidae
Source: PLoS One. 2018 May 16;13(5):e0197524. doi: 10.1371/journal.pone.0197524 (PMC5955544; doi:10.1371/journal.pone.0197524)

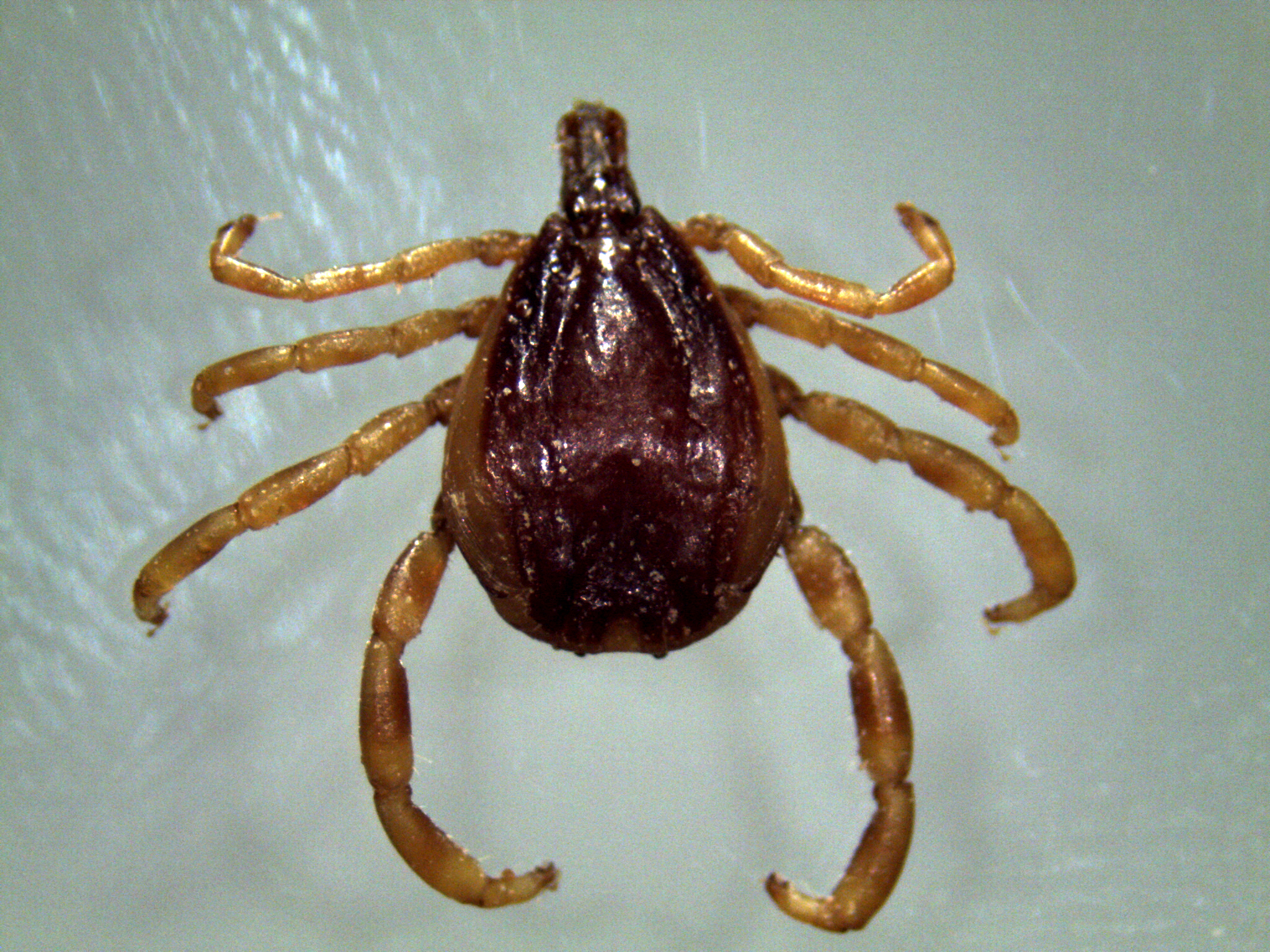

Supplement: S1 Fig — (JPG) [file pone.0197524.s001.jpg]

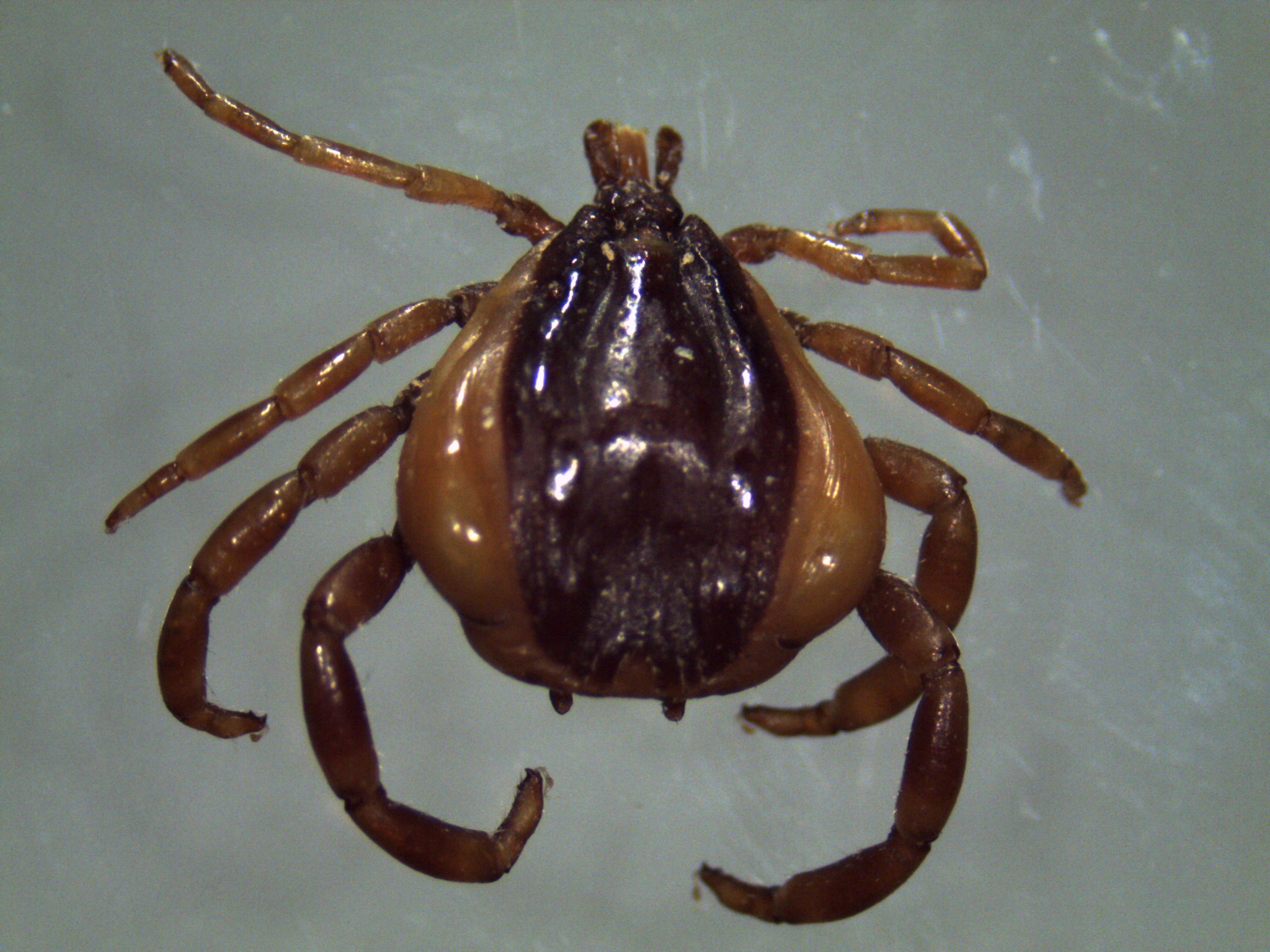

Supplement: S2 Fig — (JPG) [file pone.0197524.s002.jpg]

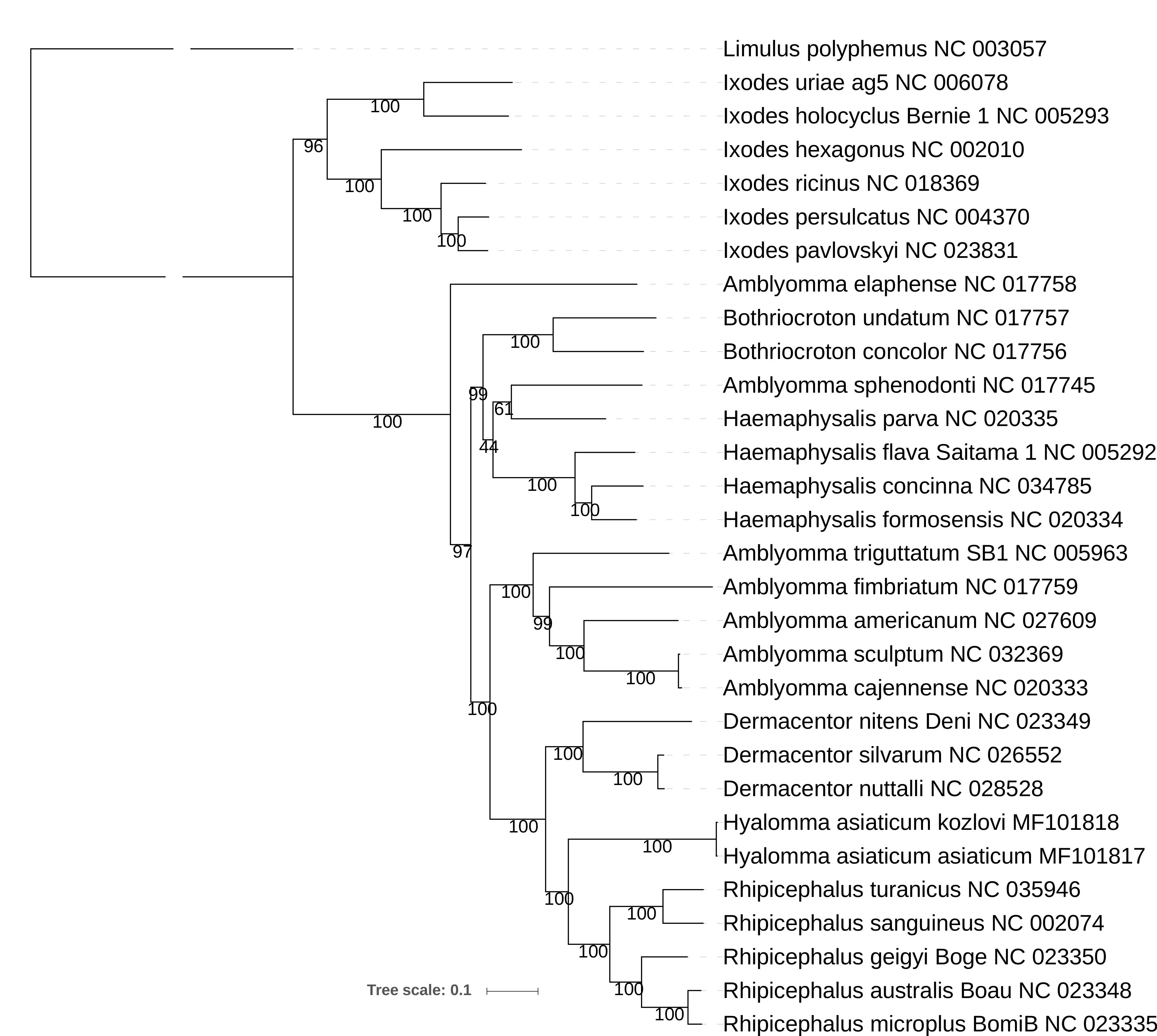

Supplement: S3 Fig — Phylogenetic dendrogram was constructed using nucleotide sequences of almost complete 29 available Ixodidae mitogenomes. Maximum Likelihood analysis was conducted using a homogeneous evolutionary model implemented in RaxML. The branch of the outgroup, Limulus polyphemus, has been shortened. Scale bar corresponds to the estimated number of substitutions per site. Bootstrap values are shown next to the corresponding nodes. GenBank accession numbers are shown next to species names, with full details available in the S1 File (supplementary data). (TIF) [file pone.0197524.s003.tif]
